# Prognostic impact of persistent postoperative neutrophil-to-lymphocyte ratio elevation 1 year after colorectal cancer surgery

**DOI:** 10.1007/s13304-025-02286-y

**Published:** 2025-06-25

**Authors:** David Ortíz-López, Joaquín Marchena-Gómez, Yurena Sosa-Quesada, Manuel Artiles-Armas, Beatriz Arencibia-Pérez, Julia Gil-García, Eva Nogués-Ramía, Cristina Roque-Castellano

**Affiliations:** Coloproctology Unit. Department of General and Digestive Surgery, Hospital Universitario de Gran Canaria Dr. Negrín, Universidad de Las Palmas de Gran Canaria, Barranco la Ballena s/n, 35310 Las Palmas de Gran Canaria, Las Palmas Spain

**Keywords:** Colorectal cancer, Inflammatory status, Neutrophil-to-lymphocyte ratio, Overall survival, Disease-free survival

## Abstract

Colorectal cancer (CRC) remains a major cause of cancer-related mortality despite advances in screening and treatment. Inflammation plays a key role in tumor progression, with the neutrophil-to-lymphocyte ratio (NLR) emerging as a potential prognostic marker. While preoperative NLR is a well-established predictor of survival, its prognostic value 1 year postoperatively remains underexplored. This study aims to evaluate the prognostic significance of NLR 1 year after curative CRC surgery, identify factors associated with its elevation, and assess its impact on survival and recurrence. A retrospective analysis was conducted on 788 patients who underwent curative-intent CRC surgery between 2015 and 2022. NLR was assessed preoperatively and 1 year postoperatively, using a cutoff of 3.3. Patients were categorized into four subgroups: “Low-Low”: NLR ≤ 3.3 pre- and postoperatively; “Low–High”: NLR ≤ 3.3 preoperatively but > 3.3 postoperatively; “High-Low”: NLR > 3.3 preoperatively but ≤ 3.3 postoperatively, and “High-High”: NLR > 3.3 at both time points. Survival analysis was performed using Cox regression. Postoperative NLR values were significantly lower than preoperative levels (median: 2.8 vs. 4.1, p < 0.001). An elevated post-NLR (> 3.3) correlated with poorer survival and higher recurrence rates. The “Low–High” group exhibited the worst prognosis, with a 5-year survival rate of 42.6% compared to 79.8% in the “Low-Low” group. Multivariate analysis confirmed post-NLR > 3.3 as an independent predictor of worse survival (HR: 3.49; 95%CI 2.41–5.04). Persistently elevated NLR 1 year after CRC surgery is associated with worse survival and higher recurrence. Routine postoperative NLR monitoring may help identify high-risk patients for closer follow-up and early intervention.

## Introduction

Despite advancements in screening and therapeutic strategies, colorectal cancer (CRC) remains a leading cause of cancer-related mortality worldwide. In the United States, CRC is the third most frequently diagnosed malignancy and the second leading cause of cancer-related death overall. Notably, it is the primary cause of cancer mortality in men under 50 years of age [1]. It has been reported that the mean number of years of potential life lost by colorectal cancer may exceed 15 years [2].

It is well known that tumor stage, high levels of serum Carcinoembryonic antigen (CEA), and carbohydrate-antigen 19–9 (CA 19.9) tumor markers, as well as certain genetic biomarkers [3] are good predictors of survival. There is also increasing evidence, suggesting that systemic inflammation is closely related to the pathogenesis, growth, and metastatic potential of CRC [4–6]. This is why in recent years, a series of inflammatory markers [7] have been added as predictors of poor outcome in CRC patients. Of these, the best known and most valued is the Neutrophil-To-Lymphocyte ratio (NLR) [8]. Several studies employing propensity score analysis [9, 10], as well as a series of published meta-analyses and systematic reviews [11–15], have demonstrated an association between elevated preoperative NLR and patient survival in CRC.

However, the progression of the inflammatory state following curative surgery for colorectal cancer and its prognostic implications remain less extensively studied. It is not yet clearly established how inflammatory markers evolve postoperatively—whether they decrease, increase, or remain stable. The long-term outcomes associated with persistently elevated NLR after surgery also remain poorly understood. Furthermore, it is unclear what occurs in patients whose inflammatory markers were initially within normal ranges but become elevated after 1 year of follow-up. While some studies have investigated this issue, their findings have been inconsistent.

This study aimed to assess the characteristics and prognostic implications of patients with elevated NLR 1 year after they underwent surgery for colorectal cancer. Additionally, to determine the factors influencing NLR elevation and to evaluate the implications of postoperative changes in this marker. We hypothesized that the persistence of an elevated NLR is a sign of poor prognosis in patients operated on for CRC with curative intent.

## Methods

### Study design and participants

This is an observational, longitudinal study from a cohort of 935 patients consecutively operated on for colorectal cancer between 2015 and 2022 at our institution. The setting was a tertiary referral center that serves a population of approximately 400,000 inhabitants.

*Inclusion criteria* patients diagnosed with colorectal cancer undergoing elective surgery with curative intent who had survived at least 1 year after surgery and had undergone a blood test at 1 year, including a full blood count. All included stage IV cases met strict criteria for curative resection, defined as complete removal of the primary tumor and all detectable metastatic lesions.

*Exclusion criteria* patients with colorectal cancer in whom palliative surgery was performed, patients with complicated colorectal cancer requiring urgent surgery, and those with incomplete postoperative histories and/or follow-up. The number and characteristics of the excluded patients were not collected.

The study was approved by the center's Clinical Research and Ethics Committee (code: 2020-279-1).

### Management of the patient

A colorectal surgeon initially evaluated patients. The preoperative diagnosis of colorectal cancer was consistently established through colonoscopy and biopsy. Staging studies included thoraco-abdomino-pelvic CT scans and/or pelvic MRI, routine blood tests, tumor markers CEA and CA 19.9, and additional investigations as required based on the patient’s underlying condition. All patients underwent mechanical bowel preparation and received antibiotic prophylaxis, orally and intravenously, before surgery. Surgical procedures were performed by a specialist colorectal surgeon.

Definitive postoperative diagnosis was based on histological examination of the resected specimen, following the criteria outlined in the 8th edition of the American Joint Committee on Cancer (AJCC) staging system[16].

The decision to administer neoadjuvant and/or adjuvant chemotherapy was guided by the protocols established by the hospital's Multidisciplinary Colorectal Tumor Board. Patients with rectal cancer classified as T3 or T4 and/or with lymph-node involvement received neoadjuvant chemoradiotherapy. Similarly, patients with stage IV colorectal cancer deemed potentially curable also underwent neoadjuvant chemotherapy. Patients with high-risk stage II and stage III colon cancer were offered standard adjuvant chemotherapy. Stage IV patients included in the study were all treated with curative intent: resection of the primary tumor and removal of metastases.

### Follow-up

Follow-up was conducted through blood tests including tumor markers every three months for the first two years, then every 6 months up to 5 years. Additionally, an annual thoraco-abdominal CT scan was performed, along with a colonoscopy at 1 year and 4 years post-surgery. Neoplasm recurrence was defined as the detection of a local recurrence on endoscopy or the presence of regional or distant metastasis on radiologic studies and/or reoperation.

### Data collection and definitions

Data were collected from a database in which patients were prospectively included. The following variables were analyzed:

### Demographic and clinical data

Age, sex, body mass index (BMI), and comorbidity measured by the Charlson Comorbidity Index (CCI) were recorded. The CCI was calculated preoperatively for each patient and includes 19 comorbidities, each assigned a weight of 1, 2, 3, or 6 based on their presence or absence. The total score ranges from 0 to 37 points [17]. A score of 0 typically indicates no comorbidity, while scores > 4 are considered indicative of severe comorbidity.

### Data laboratory

The baseline NLR was calculated from a blood sample obtained immediately before surgery. The postoperative NLR (Post-NLR) was derived from a blood sample taken 1 year after surgery in patients who could complete this follow-up period.

The NLR was calculated using the formula: absolute neutrophil count (number/μL) /absolute lymphocyte count (number/μL). High NLR values were considered to reflect a high inflammatory state.

NLR was analyzed both as a continuous variable and as a categorical variable. A cut-off value of 3.3 was applied, as determined by the Youden index [18] from a previous study on the same patient cohort [9]. This index identified the NLR value with the highest sensitivity and specificity on the ROC curve for predicting patient survival. The cutpoints were “ ≤ 3.3” for “low NLR” and > 3.3 for “high NLR”.

### Tumor localization and histopathological features

Tumor localization was categorized as colon cancer or rectal cancer based on the affected segment. Tumor staging was determined using the final histopathological report of the resected specimen and classified according to the 8th edition of the American Joint Committee on Cancer (AJCC) TNM staging system[16] (ypTNM) into stages I, II, III, and IV. Additionally, the presence of lymphovascular and/or perineural invasion was collected.

### Surgical data

Surgical procedure, surgical approach, postoperative complications according to Clavien–Dindo classification [19], and operative mortality were recorded. Operative mortality was defined as any death occurring within 30 days of surgery or any subsequent death that was determined to be a direct consequence of a postoperative complication. The variable Clavien–Dindo classification was categorized as no complications (grade 0) vs minor complications (grades I–II) vs severe complications (grades III–V).

### Neo and/or adjuvant therapy

It was recorded whether patients had received neoadjuvant chemotherapy and/or radiotherapy or adjuvant chemotherapy.

### Study groups

First, the sample was divided into two major groups according to post-NLR levels: patients with post-NLR ≤ 3.3 and patients with post-NLR > 3.3. Similarly, to assess the significance of the changes in NLR between the preoperative and 1-year postoperative values, patients were divided into 4 subgroups according to the evolution of the observed NLR values [20–22]:

(I) Normal group: pre- and postoperatively low inflammatory state (baseline NLR ≤ 3.3/post-NLR ≤ 3.3). Low preoperative values to low postoperative values *(low–low)*.
(II) Normalized group: preoperatively high but postoperatively low inflammatory state (baseline NLR > 3.3/post-NLR ≤ 3.3). High preoperative values to low postoperative values *(high–low)*.
(III) Exacerbation group: preoperatively low inflammatory state but postoperatively high inflammatory state (baseline NLR ≤ 3.3/post-NLR > 3.3). Low preoperative values to high postoperative values *(low–high)*.
(IV) Elevated group: persistently high inflammatory state (baseline NLR > 3.3/post-NLR > 3.3). High preoperative values to high postoperative values *(high–high).*

### Output measures

NLR 1 year after surgery (post-NLR), global survival time, and disease-free survival time were the output variables. Survival time was defined as the interval from the curative resection to death or censoring. Disease-free survival time was defined as the period between surgery and the detection of tumor recurrence.

### Statistical analysis

Data analysis was performed using the statistical software package SPSS version 29.0 for Windows (IBM Corporation, Armonk, NY, USA). Graphics were performed using the software Jamovi version 2.3 (The Jamovi Project, 2022).

### Descriptive analysis

Categorical variables were expressed as frequencies and percentages. Numerical variables were reported as mean (± standard deviation) and/or median (interquartile range), depending on whether or not they followed a normal distribution. Kaplan–Meier method was used to construct survival curves.

### Univariate analysis

First, we assessed whether there were significant differences between the values of baseline NLR and post-NLR. For this purpose, we employed the Wilcoxon test, a non-parametric statistical method designed for the comparison of two related samples.

A univariate analysis was then performed analyzing possible differences between patients with a high postoperative inflammatory status (post-NLR > 3.3) and patients with a low postoperative inflammatory status (post-NLR ≤ 3.3) concerning a series of predictor variables. This analysis allowed us to know which factors were related to postoperative NLR elevation and which variables could behave as confounding factors in the multivariate analysis of survival. Proportions were compared using the Chi-squared test when applicable or Fisher’s exact test when not. For numerical variables, the Student’s t test or Mann–Whitney U test was employed, depending on whether or not the data followed a normal distribution.

### Survival analysis

The log-rank test was used to compare the survival curves of patients with high and low postoperative inflammatory status. Likewise, it was employed to compare the survival curves of the four subgroups classified according to the changes in their preoperative and postoperative NLR levels.

### Multivariate analysis

A Cox regression model was performed to identify independent risk factors for long-term survival after 1 year of surgery in patients with elevated post-NLR. Predictor variables included post-NLR adjusted for all those variables that were statistically significant and clinically relevant in the univariate analysis comparing the two groups (post-NLR ≤ 3.3 vs. post-NLR > 3.3).

A *p* value < 0.05 was considered statistically significant. Hazard ratios (HR) and 95% confidence intervals (95% CI) were calculated for significant variable associations.

## Results

### Patient characteristics

Of the initial 935 patients, 788 patients met the complete inclusion criteria. All of them were followed up at least 1 year after surgery, with a mean follow-up after surgery of 4.2 years. Fifty-two patients had died during the first year of follow-up. Operative mortality was 1% (9 patients). In addition to 30-day mortality, we observed 3 additional deaths between 30 and 90 days postoperatively, totaling a 90-day mortality rate of 1.3%. The remaining cases did not reach 1 year of follow-up, did not have a blood sample determination 1 year after surgery, or had an acute episode of intercurrent pathology at that time that could alter the neutrophil–lymphocyte ratio.

Among the 788 patients included in the final study sample, 483 (61.3%) were men and 305 (38.7%) women (*p* < 0.001), median age 69 years (IQR: 62.0–76.0).

The tumor was located in the colon in 554 (70.3%) patients and in the rectum in 234 (29.7%) patients. There were 272 (34.5%) right colectomies, 64 (8.1%) left colectomies, 171 (21.7%) sigmoidectomies, 212 (26.9%) anterior rectal resections, 29 (3.7%) segmental resections, 7 (0.9%) combined resections, 20 (2.5%) abdominoperineal amputations, and 13 (1.6%) total colectomies. Regarding the surgical approach, 218 (27.7%) patients underwent open surgery, 471 (59.8%) underwent laparoscopic surgery, and 99 (12.6%) underwent robotic surgery. According to the Clavien–Dindo classification, 149 (18.9%) patients experienced minor complications, while 102 (12.9%) suffered severe complications. Anastomotic leakage was recorded in 42 (5.3%) cases. Neoadjuvant therapy was administered predominantly in rectal cancer cases. A minority of colon cancer patients with potentially resectable metastases also received neoadjuvant chemotherapy (7 patients).

During follow-up from the first year after surgery, 122 (15.5%) patients died, while 666 (84.5%) remained alive. At the same time, 139 (17.6%) recurrences were diagnosed during the follow-up of this sample of patients.

The remaining baseline characteristics of the sample are detailed in the left column of Table 2.

### Differences between baseline NLR and post-NLR

**Fig. 1:**
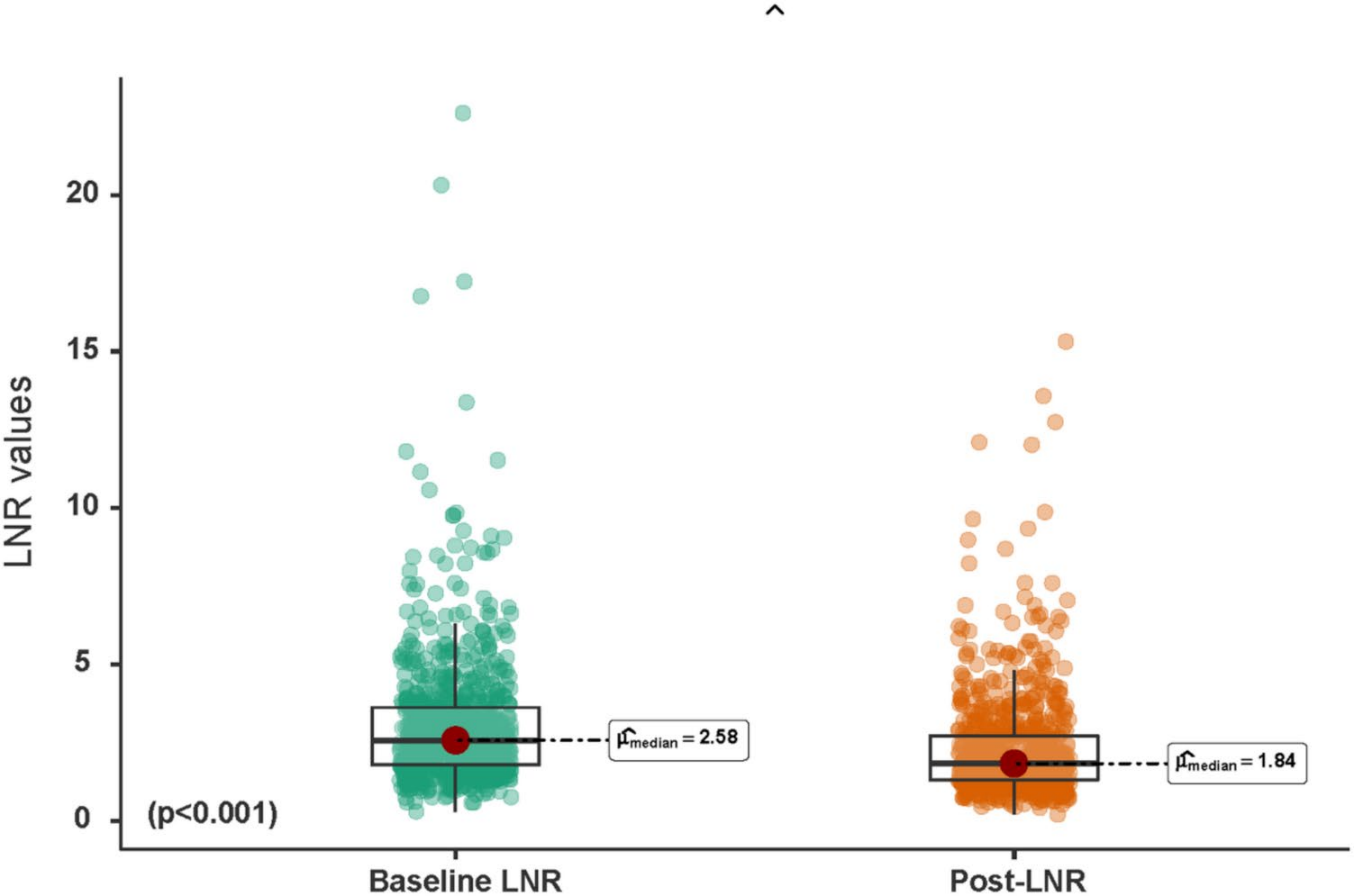
Differences observed between baseline NLR and post-NLR (1 year after surgery) as continuous variables (p < 0.001) (Wilcoxon test)

The median baseline NLR was 2.58 (IQR: 1.80–3.63), and the median post-NLR was 1.84 (IQR: 1.30–2.72). These differences were statistically significant (*p* < 0.001) (Fig. 1). Regarding the established cut-off point 3.30, 251 patients (31.9%) had an elevated NLR preoperatively, and 131 (16.6%) patients maintained elevated NLR postoperatively. These differences were also significant (*p* < 0.001).

**Fig. 2:**
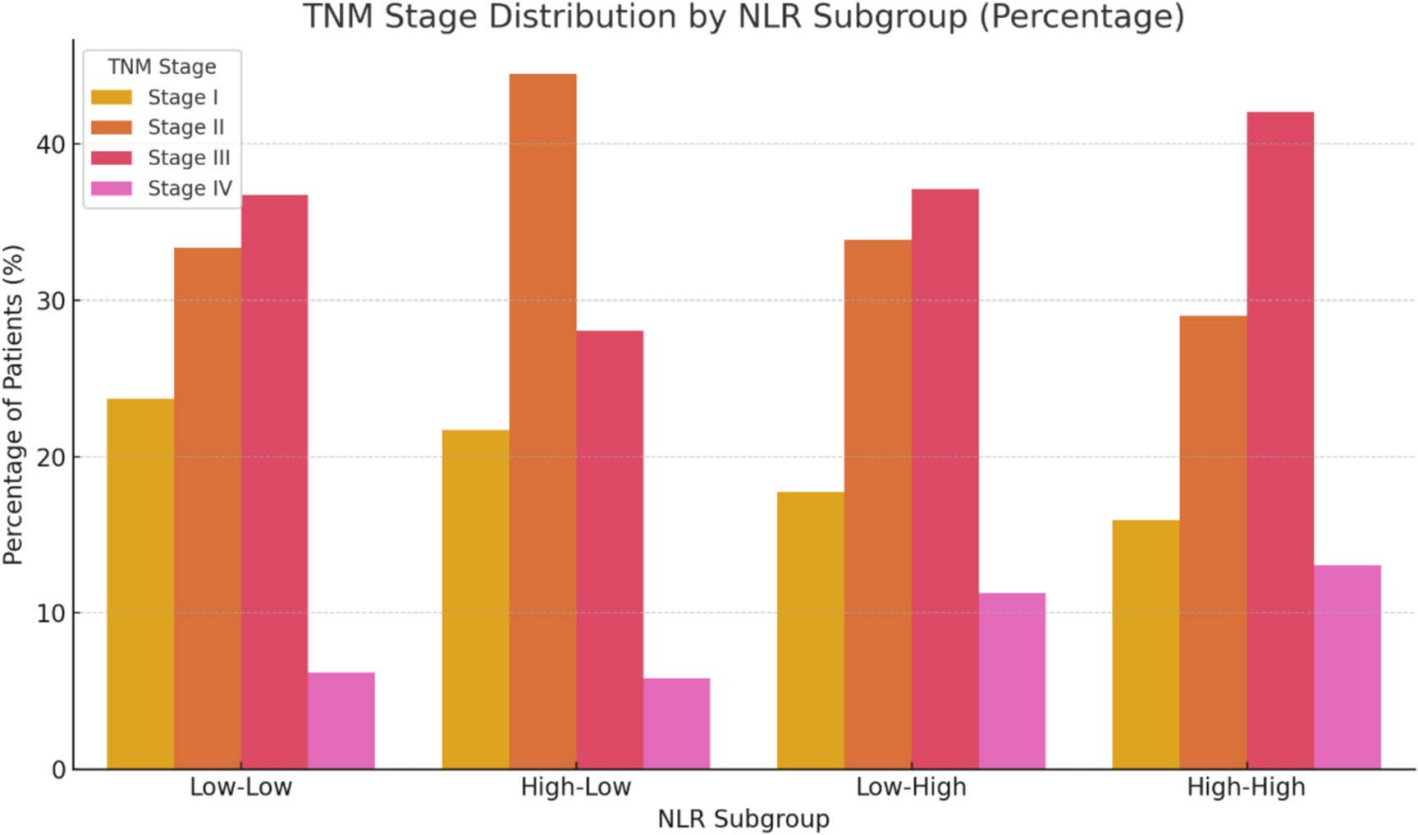
Distribution of TNM stages across postoperative NLR subgroups. A linear association was observed between subgroup classification and tumor stage (*p* = 0.028), with higher post-NLR values associated with more advanced disease stages

Regarding the four subgroups, Table 1 presents the frequencies of the various possible combinations according to the pre- and post-NLR values. Most patients (59.4%) exhibited low NLR levels before and after surgery. Regarding tumor stage, a linear association was observed between NLR subgroups and TNM stage (*p* = 0.028), with subgroups exhibiting elevated post-NLR values more frequently presenting with advanced-stage disease (Fig. 2). Stage IV patients were distributed across the four NLR subgroups, with the majority falling into the high–high group.

**Table 1:** Frequency distribution of the different subgroups according to changes observed between preoperative and postoperative NLR values

Subgroups (pre-NLR/post-NLR)	Baseline NLR	Post-NLR	n (%)	TNM stage by subgroups n (%)
Normal: pre-NLR ≤ 3.3/post-NLR ≤ 3.3	Low	Low	468 (59.4)	I: 111 (23.7) II: 156 (33.3) III: 172 (36.8) IV: 29 (6.2)
Normalized: pre-NLR > 3.3/post-NLR ≤ 3.3	High	Low	189 (24.0)	I: 41 (21.7) II: 84 (44.4) III: 53 (28.0) IV: 11 (5.8)
Exacerbated: pre-NLR ≤ 3.3/post-NLR > 3.3	Low	High	62 (7.9)	I: 11 (17.7) II: 21 (33.9) III: 23 (37.1) IV: 7 (11.3)
Elevated: pre-NLR > 3.3/post-NLR > 3.3	High	High	69 (8.8)	I: 11 (15.9) II: 20 (29.0) III: 29 (42.0) IV: 9 (13.0)

### Relationship of pre- and post-NLR with recurrence and survival

Both baseline NLR (*p* = 0.005; HR: 1.09, CI95%: 1.03–1.16) and post-NLR (*p* < 0.001; HR: 1.28, CI95%: 1.21–1.37) were significantly associated with long-term survival. At the end of follow-up, 580 (88.3%) of patients with post-NLR ≤ 3.3 and 86 (65.6%) of patients with post-NLR > 3.3 were alive. These differences were statistically significant (*p* < 0.001; OR: 3.94, 95%CI: 2.56–6.10).

**Fig. 3:**
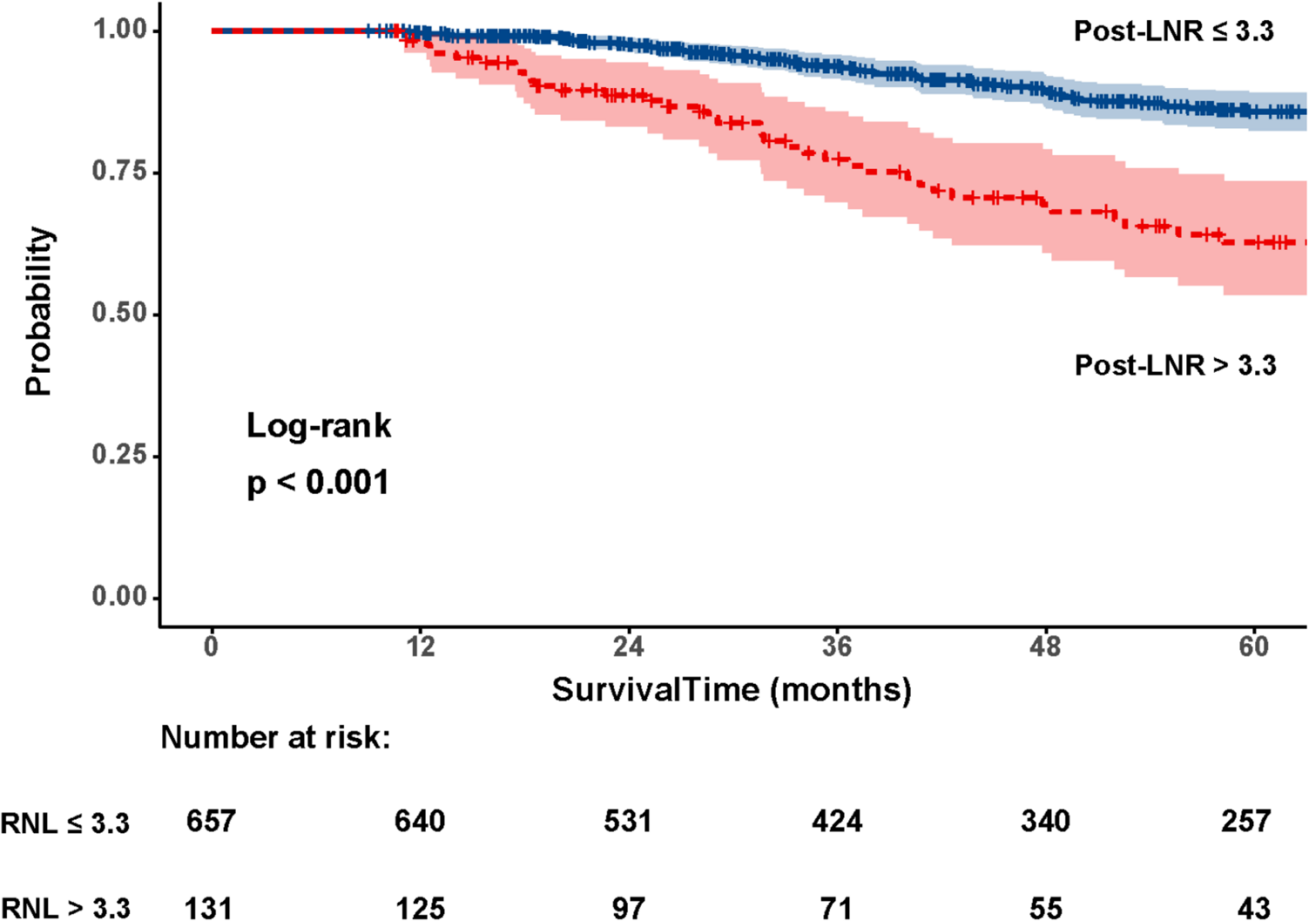
Actuarial Kaplan–Meier survival analysis for patients with post-NLR ≤ 3.3 versus post-NLR > 3 (p < 0.001). Log-Rank test

In terms of survival, patients with post-NLR ≤ 3.3 had a mean survival of 88.5 months (95%CI 86.3–90.6) while patients with post-NLR > 3.3 had a mean survival of 68.7 months (95CI 62.5–74.9) (Fig. 3). Because of the number of censored cases, median survival could not be calculated. Among patients with post-NLR ≤ 3.3, the probability of being alive at 1, 3, and 5 years was 99.7%, 93.8%, and 85.7%, respectively. In patients with post-NLR > 3.3, the probability of being alive at 1, 3, and 5 years was 99.2%, 77.4%, and 62.7%, respectively. These differences were statistically significant (*p* < 0.001; HR: 3.49, 95%CI 2.41–5.04).

**Fig. 4:**
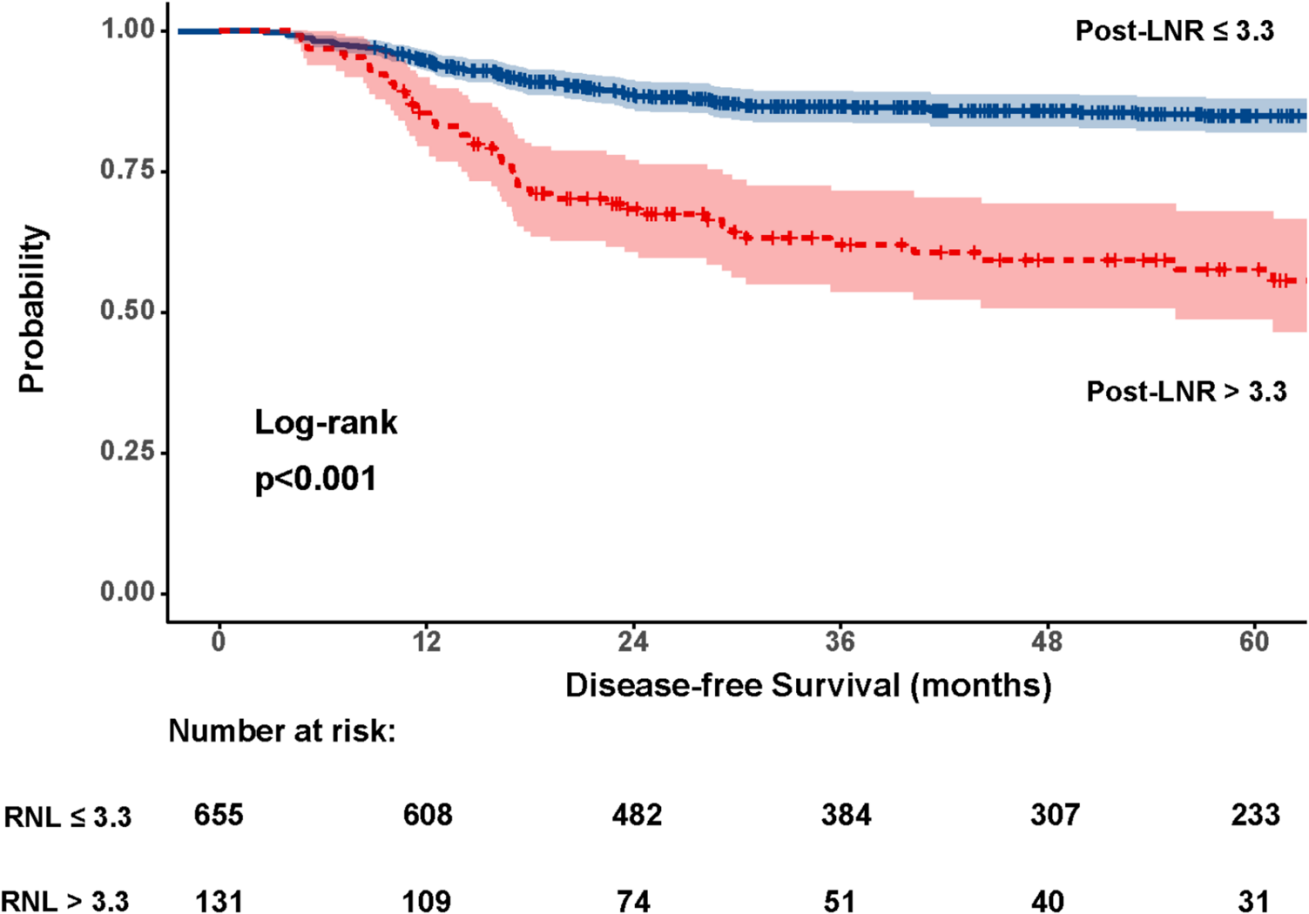
Disease-free survival analysis for patients with post-NLR ≤ 3.3 versus post-NLR > 3 (p < 0.001). Log-rank test. Note: Two patients developed a recurrence just before one-year of follow-up

Similarly, recurrence rates were significantly higher in patients with post-NLR > 3.3 (38.2%) compared to those with post-NLR ≤ 3.3 (13.5%) (*p* < 0.001; HR: 3.84, 95%CI 2.65–5.56) (Fig. 4).

**Fig. 5:**
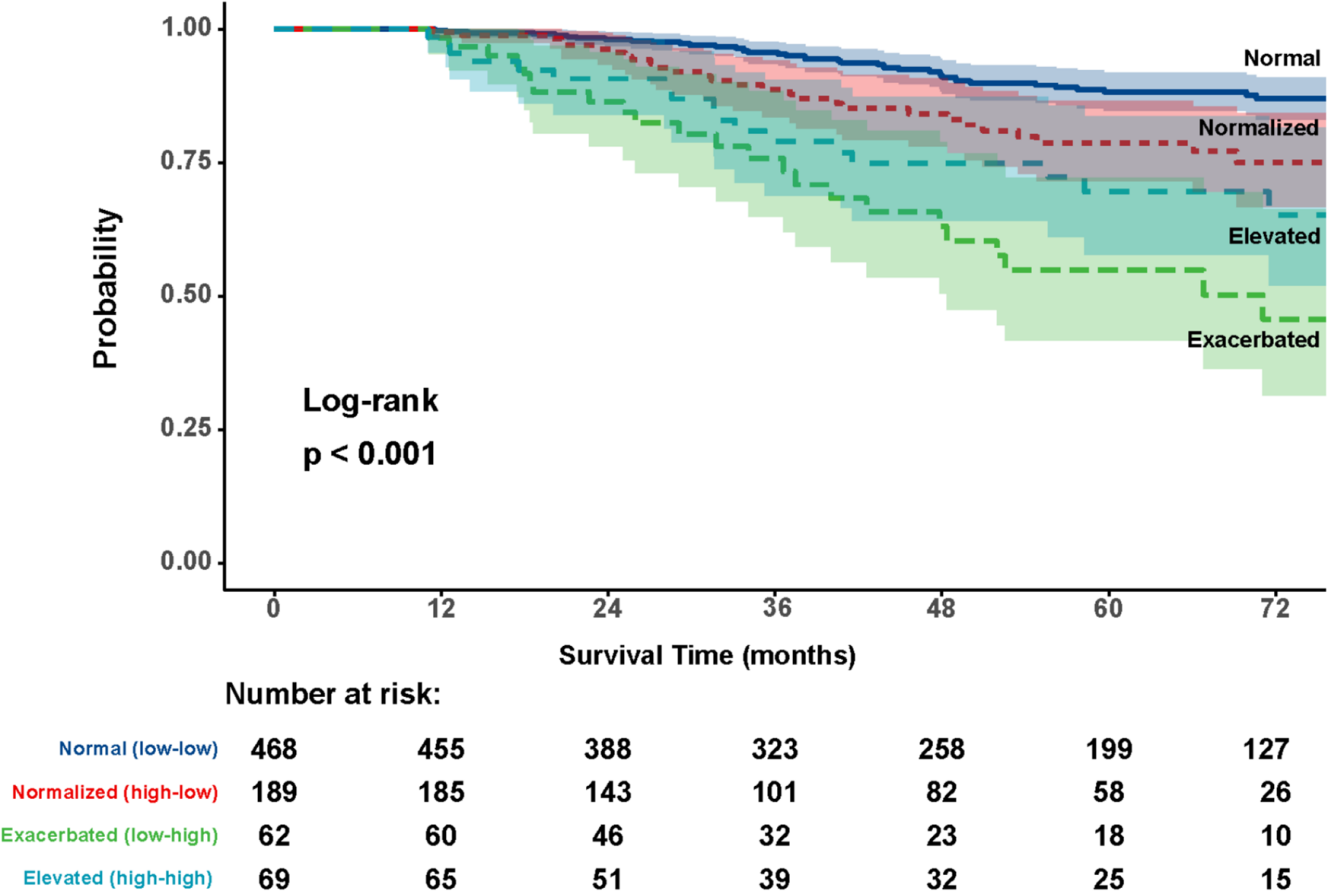
Survival curves of the different subgroups according to NLR changes observed between before and after colorectal cancer surgery. Log-rank test

When stratifying by NLR evolution, we observed significant differences between the 4 subgroups (*p* < 0.001) (Fig. 5). Patients with an NLR ≤ 3.3 who still had a low NLR 1 year after surgery had the best prognosis, with a probability of being alive at 5 years of 88.3%. In contrast, patients with a preoperative NLR ≤ 3.3 who 1 year after surgery had NLR values > 3.3 had a worse prognosis, with a probability of being alive at 5 years of only 54.9%.

### Factors associated with NLR at 1 year after surgery

Table 2 shows the variables that were associated with NLR values at 1 year after surgery and that could act as confounders in the analysis of survival. We highlight the comorbidity measured by the Charlson Index (*p* < 0.001; OR:1.25, 95%CI 1.11–1.40), the baseline NLR values both as a continuous variable (*p* < 0.001; OR: 1.27; 95%CI 1.16–1.39) and as a categorical variable (*p* < 0. 001; OR: 2.23, 95%CI 1.52–3.26), rectal location (*p* < 0.001; OR: 3.13, 95%CI 2.26–4.88), tumor stage (*p* < 0.001; OR: 1.36, 95%CI 1.10–1.70), and neoadjuvant therapy (*p* < 0.001; OR: 3.84, 95%CI 2.52–5.85).

**Table 2:** Univariate analysis of persistent elevation of NLR 1 year after surgery. *Statistically significant. OR: Odds Ratio. 95% CI: 95% Confidence Interval

	Total N (%) 788 (100)	Post-NLR ≤ 3.3 N (%) 657 (83.4)	Post-NLR > 3.3 N (%) 131 (16.6)	p	OR (95% CI)
Age Median (IQR)	69.0 (62.0–76.0)	69.0 (62.0–76.0)	70.0 (57.0–77.0)	0.265	0.99 (0.97–1.01)
Sex:					
Men	483 (61.3)	394 (60.0)	89 (67.9)	0.088	0.71 (0.47–1.05)
Women	305 (38.7)	263 (40.0)	42 (32.1)		
Charlson score Median (IQR)	3.0 (2.0–3.0)	2.0 (2.0–3.0)	3.0 (2.0–4.0)	< 0.001*	1.25 (1.11–1.40)
BMI Median (IQR)	27.0 (24.0–29.4)	27.0 (24.0–29.4)	27.0 (24.0–29.0)	0.896	1.00 (0.96–1.05)
Basal NLR Median (IQR)	2.58 (1.83–3.67)	2.46 (1.73–3.45)	3.25 (2.24–4.83)	< 0.001*	1.27 (1.16–1.39)
Basal NLR categorized:					
≤ 3.3	537 (68.1)	468 (71.2)	69 (52.7)	< 0.001*	2.23 (1.52–3.26)
> 3	251 (31.9)	189 (28.8)	62 (47.3)		
Tumor location					
Colon	554 (70.3)	492 (74.9)	62 (47.3)	< 0.001*	3.13 (2.26–4.88)
Rectum	234 (29.7)	165 (25.1)	69 (52.7)		
Tumor stage					
I	174 (22.1)	152 (23.1)	22 (16.8)	0.005*	1.36 (1.10–1.70)
II	281 (35.7)	240 (36.5)	41 (31.3)		
III	277 (35.2)	225 (34.2)	52 (39.7)		
IV	56 (7.1)	40 (6.1)	16 (12.2)		
Nodal stage					
N0	553 (70.2)	467 (71.1)	86 (65.6)	0.215	1.29 (0.86–1.92)
N +	235 (29.8)	190 (28.9)	45 (34.4)		
Lymphovascular invasion					
No	548 (69.5)	456 (69.4)	92 (70.2)	0.852	0.96 (0.64–1.45)
Yes	240 (30.5)	201 (30.6)	39 (29.8)		
Perineural invasion					
No	617 (78.3)	515 (78.4)	102 (77.9)	0.894	1.03 (0.66–1.62)
Yes	171 (21.7)	142 (21.6)	29 (22.1)		
Postoperative complications:					
No	537 (68.1)	456 (69.4)	81 (61.8)	0.074	1.25 (0.98–1.61)
Minor	149 (18.9)	121 (18.4)	28 (21.4)		
Severe	102 (12.9)	80 (12.2)	22 (16.8)		
Neoadjuvant therapy					
No	654 (83.0)	571 (86.9)	83 (63.4)		
Yes	134 (17.0)	86 (13.1)	48 (36.6)		
Adjuvant therapy					
No	444 (56.3)	377 (57.4)	67 (51.1)	0.189	1.29 (0.88–1.87)
Yes	344 (43.7)	280 (42.6)	64 (48.9)		
Recurrence in follow-up:					
No	649 (82.4)	568 (86.5)	81 (61.8)	< 0.001*	3.94 (2.60–5.98)
Yes	139 (17.6)	89 (13.5)	50 (38.2)		
Dead in follow-up					
No	666 (84.5)	580 (88.3)	86 (65.6)	< 0.001*	3.94 (2.56–6.10)
Yes	122 (15.5)	77 (11.7)	45 (34.4)		

### Multivariate analysis

**Fig. 6:**
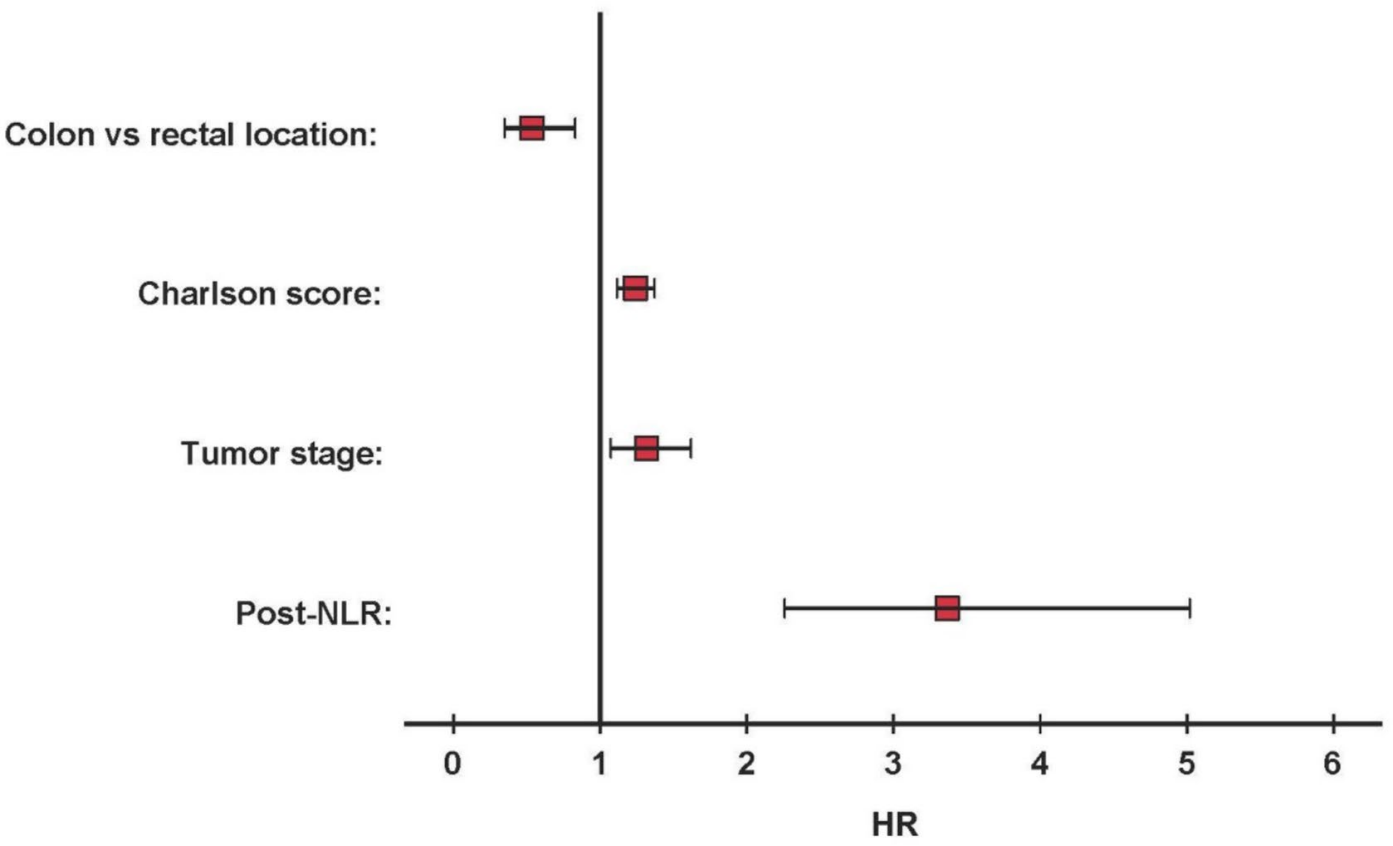
Forest plot for survival of patients with colorectal cancer 1 year after curative resection based on multivariate Cox proportional hazard model

The following variables were entered in a Cox regression model: post-NLR (≤ 3.3 vs > 3), comorbidity measured by Charlson score, tumor stage, and rectal location. Collinearity was detected between variable rectal location and neoadjuvant therapy, so neoadjuvant therapy variable was not included. Post-RNL (*p* < 0.001; HR: 3.37, 95%CI 2.26–5.02), tumor stage (*p* = 0.009; HR: 1.32, 95%CI 1.07–1.62), comorbidity (*p* < 0.001; HR: 1.24, 95%CI 1.11–1.37), and rectal location (*p* = 0.005; HR: 0.54, 95%CI 0.35–0.83) remained independent predictors of long-term survival after 1 year of surgery (Fig. 6).

## Discussion

The results of this study, using NLR as an inflammatory marker, confirm the importance of the patient's inflammatory status in colorectal cancer prognosis 1 year after surgery. Based on the results obtained, patients with post-NLR > 3.3 are four times more likely to experience recurrence or death. Multivariate analysis identified post-NLR as an independent prognostic factor, even after adjusting for tumor stage, comorbidity, and tumor location. Therefore, the presence of post-NLR levels > 3 1 year after surgery defines a population of patients operated on for CRC at very high risk of recurrence and death during follow-up. Other authors have reached the similar conclusions with different NLR cut-off points [20, 22–24], although in one study, NLR was not subject to significant overall change from the pre- to postoperative period [24].

Both preoperative and postoperative NLR are useful prognostic factors for survival and recurrence. However, most of the studies suggest that post-NLR is a stronger predictor [20]. This supports the idea that tumor removal may enhance immune function, making posttreatment NLR a reliable indicator of future recurrence [25].

Post-NLR levels have been assessed either as absolute values at a specific time after surgery [22, 25, 26], or by considering the dynamics of longitudinal changes in their values at different postoperative times [21, 23, 27].

We believe that one of the most interesting aspects in these cases is to evaluate changes in inflammatory status between pre- and postoperative periods, 1 year after surgery in our study. We found that the type of variation observed after curative CRC resection in the inflammation-based prognostic marker NLR values has a clear prognostic impact on tumor recurrence and long-term survival.

Our study demonstrates that, after resection of colorectal neoplasia, in most patients, the post-LNR values are maintained (normal subgroup: “low-low”) or decrease significantly with respect to baseline NLR (normalized subgroup: “high-low”), indicating a good prognosis. However, those with persistently high NLR (elevated group: “high-high”) or an increase from low to high levels (elevated group: “low–high”) after 1 year from surgery have a worse prognosis, as reflected in survival curves.

These findings are consistent with those reported by other authors [21] with different NLR cut-off points and different blood sampling times during follow-up. However, one study [20] reported that the exacerbation subgroup (“low–high”) had a similar prognosis to the persistently normal subgroup (“low-low”). Shibutani et al. [22] also found that the “low-low” subgroup had a better prognosis in survival compared to the remaining subgroups, but they did no find significant differences among these other subgroups.

Therefore, changes in NLR may reflect treatment efficacy and its impact on survival [21, 23, 25]. However, three questions arise regarding the use of post-NLR as a prognostic factor in patients operated on by colorectal cancer.

First, whether NLR is the most appropriate inflammatory marker in these situations. Several inflammatory markers have been proposed for this purpose, including some prognostic scales [28], but many comparative studies have demonstrated its superiority to other markers [8, 29]. We believe that the NLR is the most accessible and cost-effective option.

Second, there were also very notable differences among the various published series regarding the timing of blood sampling to assess postoperative NLR levels: 21–56 days [21], 21–90 days [20], 1 month [22, 23, 25], between 1 and 3 months [26], between 3 and 6 months [24]. Our study used a 1-year timeframe, allowing sufficient time to minimize the influence of surgical trauma or adjuvant therapy on hematologic parameters.

Third, there is still no consensus on the optimal NLR cut-off value for CRC prognosis. Normal NLR values in healthy adults range from 0.78 to 3.53 [30], while in CRC patients, cut-offs typically range from 2 to 5 [7]. Most studies in patients with CRC have used a threshold around 3 [21, 22, 25, 26], which closely aligns with our chosen cut-off of 3.3.

The mechanism underlying and the significance of the persistent elevation of the systemic inflammatory response in patients who have undergone resection of the primary CRC (subgroup “high-high”) remain unclear. It is thought that may reflect the complex interaction between the local immune response at the tumor microenvironment and the systemic inflammatory response [21].

There is sufficient evidence of the inflammatory pathogenesis of colorectal cancer [4]. Proinflammatory systemic factors can act as initiators of carcinogenesis, and, during neoplastic transformation, tumor cells also release proinflammatory substances which contribute to creating a situation of systemic inflammation [5]. At the same time, certain substances released by tumor associated neutrophils may act to promote tumor growth [31]. On the other hand, lymphocytes, both peripheral blood and tumor infiltrating lymphocytes, play a key role in antitumor immunity [32]. In fact, the absolute lymphocyte count is assumed to reflect the degree of responsiveness of a cancer patient’s whole immune system [33]. This would explain why patients with elevated NLR levels would have a worse prognosis. Therefore, a persistently high NLR after surgery means the continuation of an environment that is favorable for recurrence.

From a clinical point of view, some authors [21, 22, 24, 25] have already suggested the more than possible association between postoperative high levels of neutrophil–lymphocyte ratio and the presence of minimal residual disease or early metastatic disease in these patients. Murray et al. [25] defined minimal residual disease as bone marrow micro-metastases and circulating tumor cells, noting that NLR only decreased in patients without these circulating cells. Their presence was associated with immune dysfunction and poorer prognosis.

However, Yasui et al. [20] suggest that persistently high inflammatory markers may reflect a patient’s intrinsic inflammatory state. To demonstrate this, they analyzed various prognostic markers, including NLR, in the “elevated” subgroup 5 years post-surgery without recurrence. Over 90% maintained high inflammation levels, though initial tumor stage data were not provided.

Our results support the hypothesis that high NLR levels 1 year after surgery often indicate undetectable disease progression. Survival curves confirm this trend. In addition, the univariate analysis revealed that patients with high post-NLR levels typically had more advanced disease. However, it is questionable whether the response to the micrometastatic lesion and the response to the primary tumor are equivalent [22].

Univariate analysis identified comorbidity, neoadjuvant therapy, and tumor location as potential confounders, alongside tumor stage. Regarding tumor stage, all included stage IV cases met stringent criteria for curative resection. These patients represent a distinct subgroup with potentially favorable long-term outcomes [34], and for this reason, they were not excluded from the study. In addition, acknowledging the potential for confounding, we included tumor stage, post-NLR, tumor location, and comorbidity as covariates in the multivariate analysis, with the intention to adjust for their possible influence on survival outcomes. Tumor stage emerged as an independent prognostic factor. These findings support the relevance of post-NLR assessment even in patients with stage IV disease.

Comorbidity was strongly associated with post-NLR and is known to predict higher mortality in cancer patients, including CRC, particularly from cardiovascular causes [35]. Comorbidity could also have influenced survival because of its relationship with the poor tolerance to chemotherapy that some of these patients would present [36]. These data were not analyzed in our study.

With respect to tumor location, it has been suggested that there is a greater inflammatory response in rectal cancer than in colon cancer, which may explain why preoperative NLR is not always an independent survival predictor in patients with rectal cancer [37]. Additionally, neoadjuvant radiotherapy, common in rectal cancer, may induce a prolonged inflammatory response, contributing to elevated post-NLR levels.

Limitations of this study include the single-center and retrospective study design. Its retrospective design may introduce selection bias and unmeasured confounding factors. Additionally, genetic status data (KRAS, BRAF, and microsatellite instability), which may play an important role in the prognosis of the neoplasm, were not analyzed. Strengths include a large, representative sample, standardized blood collection timing, and the use of a single reference point (1 year post-surgery) to assess NLR’s prognostic significance. Data were also collected prospectively, and control for potential confounders was performed by multivariate analysis.

## Conclusions

Our study underscores the prognostic value of NLR 1 year after colorectal cancer surgery. Elevated pre- and post-NLR levels correlate with poorer survival, suggesting a role for persistent inflammation in disease progression. Patients with increasing or consistently high NLR have worse outcomes. Future research should assess NLR’s role in treatment decisions and its integration into prognostic models. NLR should be routinely included in blood test reports.

## Data Availability

The data sets generated during and analyzed during the current study are available from the corresponding author on reasonable request.

## Declarations

### Permission to reproduce material from other sources

No material from other sources was included in this article.
